# Correction: Proximity-induced SuFEx increases the potency of cytosolic nucleotidase inhibitors and reveals a rare example of covalently targeted histidine

**DOI:** 10.1039/d5cb90025e

**Published:** 2025-06-12

**Authors:** Mikolaj Chrominski, Marcin Warminski, Mateusz Kozarski, Dorota Kubacka, Joanna Panecka-Hofman, Tomasz Spiewla, Mikolaj Zmudzinski, Jacek Jemielity, Joanna Kowalska

**Affiliations:** a Centre of New Technologies, University of Warsaw Banacha 2c 02-097 Warsaw Poland m.chrominski@cent.uw.edu.pl; b Division of Biophysics, Faculty of Physics, University of Warsaw Pasteura 5 02-093 Warsaw Poland jkowalska@fuw.edu.pl

## Abstract

Correction for ‘Proximity-induced SuFEx increases the potency of cytosolic nucleotidase inhibitors and reveals a rare example of covalently targeted histidine’ by Mikolaj Chrominski *et al.*, *RSC Chem. Biol.*, 2025, **6**, 942–947, https://doi.org/10.1039/d5cb00005j.

The coauthor Jacek Jemielity’s name was spelled incorrectly in the originating article, the correct author list is shown here.

The Royal Society of Chemistry apologises for these errors and any consequent inconvenience to authors and readers.

